# Comment on “A new method for treating fecal incontinence by implanting stem cells derived from human adipose tissue: preliminary findings of a randomized double-blind clinical trial”

**DOI:** 10.1186/s13287-018-0875-4

**Published:** 2018-04-25

**Authors:** Mohammed Mohammed El-Said, Sameh Hany Emile

**Affiliations:** General Surgery Department, Colorectal Surgery Unit, Mansoura Faculty of Medicine, Mansoura University, Mansoura city, Dakahlia providence Egypt

## Abstract

In the study by Sarveazad et al. adipose tissue-derived stem cells were injected to reinforce anal sphincter repair. The authors came to the conclusion that injection of stem cells during repair surgery for fecal incontinence may cause replacement of fibrous tissue, which may be a key point in treatment of fecal incontinence. The authors emphasized in their “Discussion” section that the ability of stem cells to differentiate into muscle fibers, replacing the fibrous tissue at the site of repair, is their main action, which may not be accurate. We think that healing of repaired anal sphincter begins with granulation tissue formation, which then matures into fibrous tissue that becomes infiltrated by muscle fibers from the approximated cut ends of the sphincter, resulting in regain of sphincter muscle continuity. This is supported by many experimental studies that have evaluated local injection of stem cells during sphincteroplasty in rats and shown that the injected stem cells do not differentiate into muscle fibers but may induce healing by a strong fibrous tissue. Further studies are needed to determine the main mechanism of action of mesenchymal stems cells in augmenting anal sphincter repair.

First of all we were pleased to read the article by Sarveazad et al. [1] in your prestigious journal. This study was in line with our previous study that assessed augmentation of anal sphincter repair by injecting bone marrow aspirate concentrate [2]. However, we have a few comments on the study.

In the “Background” section, the authors attributed the possible benefits of injecting human adipose-derived stem/stromal cells (hADSCs) after sphincteroplasty to a number of factors, including easier and safer access, secretion of multiple growth factors, and production of high levels of angiogenic factors in addition to differentiation into muscle fibers. However, throughout the “Discussion” and the “Conclusion” sections they emphasized that the ability of stem cells to differentiate into muscle fibers, replacing the fibrous tissue at the site of repair, is their main action, which may not be accurate.

Viewing the current literature, several experimental studies [3–5] have evaluated the mechanism of action of stem cell injection in animal models. The anal sphincters of rats were cut and repaired with and without local injection of stem cells; the rats were then sacrificed and the site of repair was examined to determine if healing occurred by fibrosis or muscle regeneration.

Pathi et al. [4] showed that stem cell injection increased matrix deposition with the formation of more dense fibrous tissue scar at the site of repair. In contrast, Lorenzi et al. [3] reported conflicting results as they showed that stem cell injection improved muscle regeneration after they found muscle fibers at the site of repair when stem cells were injected but not when stem cells were not injected. The results of Fitzwater et al. [5] appear even more confounding as they demonstrated that stem cell injection has no histological effect at the site of repair, and furthermore, at 90 days sphincter muscle continuity was regained whether stem cells were injected or not.

The key to understand these apparently conflicting results appears to be the time of examination of the site of repair. When the repair site was examined at 7 days [5], a gap of granulation tissue was present within the sphincter even if stem cells were injected. On the other hand, on examining the site of repair at 21 days [4], fibrous tissue was more dense when stem cells were injected. When the site of repair was examined at 30 days [3], muscle fibers appeared at the site of repair only when stem cells were injected, and at 90 days [5], muscle continuity was regained even if stem cells were not injected. The different methods used in these studies and the varying timing of examination of the site of sphincter repair after stem cell injection may have resulted in the disparate outcomes observed.

We think that healing of repaired anal sphincter in rats begins with granulation tissue formation, which matures into fibrous tissue that becomes infiltrated by muscle fibers from the approximated cut ends of the sphincter, resulting in regain of sphincter muscle continuity. Stem cell injection seems to only accelerate this process. Pathi et al. [4] showed that levels of lysyl oxidase and transforming growth factor β1 mRNA (both involved in extracellular matrix deposition) peak at 24 h if stem cells were injected versus 7–21 days if stem cells were not injected.

As a result of accelerated healing by stem cell injection, muscle fibers were detectable at 30 days after repair; when stem cells were not injected muscle fibers were detected at a later stage. Fitzwater et al. [5] showed that the volume of striated muscle fibers in the external sphincter at 90 days was not increased by stem cell injection, implying that injected stem cells do not differentiate into muscle fibers.

In contrast to the aforementioned experimental studies that questioned the concept of differentiation of injected stem cells into muscle fibers, evidence in the current trial supporting muscle fiber differentiation of injected stem cells was derived from non-invasive methods such as EAUS and EMG. The EMG showed action potential at the repair site in the hADSC group but not in the control group, which may be due to a stronger scar approximating the muscle ends or faster healing by fibrosis allowing infiltration of the scar tissue by muscle cells from the cut muscle ends. The optimal method to ascertain differentiation of stem cells into muscle fibers would be by histopathologic examination of a biopsy from the site of repair. However, although histopathologic examination may be more conclusive than non-invasive methods such as electromyography (EMG) and endoanal ultrasonography (EAUS), this examination is not applicable due to ethical reasons.

Overall, the exact mechanism by which stem cells improve anal sphincter repair and produce a stronger scar is still not clear. Several mechanisms were proposed, including secretion of multiple growth factors, production of high levels of angiogenic factors, and differentiation into muscle fibers or fibroblasts.

Lee et al. [6] proved that connective tissue growth factor is a potent stimulator of fibroblastic differentiation of human mesenchymal stem cells by increasing type I collagen and tenascin-C synthesis. Therefore, differentiation of human mesenchymal stem cells into fibroblasts is possible and reproducible as Lee and colleagues implied. Further studies are needed to determine the main mechanism of action of mesenchymal stems cells in augmenting anal sphincter repair.
